# An Ishmaelite

**Published:** 1888-04-28

**Authors:** Leslie Keith

**Affiliations:** Author of "The Chilcotes," "Uncle Bob's Niece," "East and West," etc.


### An Ishmaelite.

By Leslie Keith,

Author of "The Chilcotes," "Uncle Bob's Niece," "East and West," etc.

### Chapter IV.—Paying the Penalty.

Five years' penal servitude—those words meet the eye from time to time as we glance down the columns of the daily prints, and they are often, too, but unmeaning sounds to our ear. At best we say, "Poor wretch," and pass on to something of more interest. Few of us can put ourselves in the place of the victim and enter into his mental condition as that sentence of doom is pronounced over him.

In reckless despair Fred had forged his godfather's signature. Mr. Mack, too, was away from home; everything seemed to be against Fred, and he yielded to an almost irresistible temptation. He presented the cheque himself at the bank; it was a busy day—a Saturday—and the cashier paid it, but the forgery was too palpable and clumsy to escape immediate detection. Fred's was the latest of several similar attempts, and the bank had vowed vengeance against the next offender, let him be whom he might. Fred was detained on some pretext, and given in charge before he left the building. Perhaps even if his father could have stayed proceedings by a word, he would not have uttered that word. The love which he had torn out of his heart went to feed the fountains of his hate. "He is beyond redemption," he said; "he is no son of mine."

What he suffered no one ever knew. He went back to the City and to business after a single day, while Fred lay in gaol awaiting his trial. The clerks, when they looked into his face—grown hard as adamant—trembled in their shoes, but he bore himself with a terrible composure that was worse to see than his anger. When Mr. Mack, who had been down in the country, came up to town, agitated and overwhelmed with the tidings, Mr. Eastgate received him with a nod and a question as to the prospects of the spring sowing.

Mr. Mack, whose good-natured face was purple with agitation, stared at his friend with a terrible conviction that he had gone mad under this sad blow.

"Good heavens! Joseph," he said, unconsciously using the old boyish name, "you don't suppose I had anything to do with it! The bank might have saved the scandal by refusing to prosecute. It was a mere bit of spite, since the money was safe enough. I would have helped the poor lad if I had known he was in a mess. If that confounded business down at Lark Hall hadn't taken me out of town——"

"Of whom are you talking?" asked Mr. Eastgate coldly.

"Of—of Fred—of your boy—your unhappy son," faltered the other in his surprise.

"I have no son," said Mr. Eastgate, with an awful sternness that struck the visitor dumb. It was the same at home; there too he announced to the astonished servants that he had no son. He called them up to the library, and commanded that Fred's name should never be mentioned in that house again, under penalty of instant dismissal. Mrs. Eastgate's maid waited when the others had flocked out to discuss this strange order in that parliament of the servants' hall where the upstairs doings are canvassed. She waited, and braved Mr. Eastgate's gloomy glances, that she might give warning. "She could not live under the same roof with such a monster of cruelty," she said, when she rejoined her comrades below. Her mistress was ill upstairs, dying perhaps—"And if she dies, mark my words, it's murder he'll have committed," she said, "and it's he that ought to be in prison if the right people was always punished."

Fred had been a favourite with the servants for a certain winning, bright way he had, and all the sympathy was on his side. Old stories of the young man's harsh treatment as a child were recalled, exaggerated and coloured till Mr. Eastgate became a sort of Francesco Cenci in the eyes of his servants. No one pitied him or guessed at the intolerable pangs of wounded pride and rage that rent him. On the day of the trial, when Fred was condemned and sentenced, he went to the room that his son had used on his visits home. He called Nellie to come to him, and the girl obeyed in fear. He made her take down the pictures, the little nick-nacks—evidences of Fred's fine taste—his gun, his fishing-rod, and hide them out of sight: he watching her the while with stern composure. She despoiled the pretty room at his bidding, the tears falling so fast that they nearly blinded her.

"He is not dead," she said, with a timid little protest.

"Would God he were lying there dead!" said the father, looking at the bed. It was the only sign of anguish that escaped him. He rallied immediately, and said harshly to the frightened girl: "He is dead! Dead to you and me from this day. I shall never look on his face again."

Mrs. Eastgate was dangerously ill for a long time, but she rallied slowly, and began with the summer to regain a faint hold on life. The best doctors and nurses were at her service, and every aid that money could procure. Her husband went to ask for her daily before going to the City. He was attentive and civil, but she seemed almost indifferent to his inquiries. She no longer trembled or shrank in his presence; she seemed hardly to see him indeed, but her gaze went past him with a most pathetic, hungry look. The poor lady, though she was presently carried down to the drawing-room, was sadly altered and shattered, and sometimes Nellie fancied she scarcely knew what had befallen her. She did not speak of Fred, but there was always that piteous, wistful questioning in her eyes.

In the City it was said that Eastgate bore his trouble well, and with a pride that forbade commiseration; but at home he wore habitually a speechless gloom. There were no more feasts in the big house; Nellie and he dined opposite each other in unbroken silence day by day: the servants would not stay in a house where even to laugh was a crime. They went and others came, and the sad monotonous life went on till Nellie wondered sometimes if there had ever been a time when she was young and gay. Meals were spread, and prayers were read every night by the stern master in the library. Once, when in the course of his reading he came to that story of another prodigal who had strayed and came back and was forgiven, he shut the book and dismissed the astonished household: the divine compassion and tender love of that other father found no echo in his heart. Love was dead there, and hate reigned in its stead.

When Arthur heard the news he came rushing up to town, frantic with grief. He refused utterly to obey that mandate his uncle had given. "Not speak of him!" he cried, looking into the hard, dark face. "Why, I loved him; I love him still. Am I to give him up because of one slip—the work of a moment? It might be me—or you to-morrow."

Strange to say, Mr. Eastgate had no reply ready. The lad's brave air and his honest, indignant face somehow silenced him. "Ah, if this had been my son," he thought, with surging bitterness, as he looked at the boy's open, fearless face.

Arthur had more than one interview with his cousin before the trial. He moved heaven and earth to make it go in his favour, and he cheered and consoled him as well as he could, though it was all he could do to command himself.

Fred did not break down even when he sent a message to his mother; but he knew he had no chance.

"Tell—tell Nellie to forget me; and to forgive me if she can," he said falteringly, for the first time showing signs of emotion. Arthur wrung his cousin's hand as he bade him good-bye.

"We shall none of us ever forget you," he said. Fred had always been his hero; he had loved him and looked up to him with all the ardour and generosity and simplicity of boyhood, and, as he said, he loved him still. If Fred had transgressed, there was but reason the more to stick to him.

When he got back to Portland Place after that last visit, Nellie was waiting for him on the stair. Pretty Nellie had a very appealing look on her sad young face. She drew Arthur into the drawing-room.

"Did he—did he send any message to me, Arthur?"

Arthur put his hands on her shoulders and looked down into the beseeching face.

"He said you were to forget him, and to forgive him if you could, Nellie."

She blushed up a sudden hot crimson. "Forget him! Oh Arthur! I can't—I can't."

"No, Nellie"—Arthur spoke firmly—"you can't, and you won't; I should think but little of my sister if she could so easily put the dear old fellow who is our brother out of her heart. We will think of him always as our brother, and—and if we must lose him for a little, he will come back to us one day. I am going to Aunt Helen now." He broke off bruptly, a little shy, perhaps, before this display of his emotions.

"You mustn't speak to Auntie about Fred, Arthur; the doctors have forbidden it."

"The doctors!" cried Arthur, with an infinite scorn. "Don't you see that it is just this silence that is killing her?"

"They say she must not be agitated."

"Rot," said Arthur, finding a relief for his oppressed heart in this vigorous contempt; "much they know about it. She is perishing for a little sympathy, and is afraid to ask for it. That is what they have done—they have made her afraid."

He went to her room and sat down by her sofa. She scarcely noticed him at first, but when he took her hand and began courageously and cheerfully to talk of Fred, her wistful eyes fixed themselves eagerly on his face.

Arthur felt a lump rise in his throat, but he talked on bravely and brightly. The world had not soiled the freshness of his beliefs as yet; disappointment had not soured him: and he could look beyond the present with a sanguine faith and see a brightening future for Fred too. When he knelt down to whisper Fred's message in the mother's ear she put her feeble arms round him and wept as she had not wept since the fatal day. When Nellie stole in an hour later she found the room hushed and silent; the invalid peacefully asleep with her hand locked in Arthur's.

When Arthur went to Oxford it seemed to him that the air was full of memories of his cousin. The mellow tongue of Great Tom as it proclaimed the hours, the airy carillon flinging its notes from Magdalen tower, every melodious voice in that city of bells, where the hours are told to numbers, spoke to him of his cousin. The yelling crowd by the river's brim, the hoarse shouts at a 'Varsity match—he caught himself listening for Fred's voice there too. He failed, to his great disappointment, to inherit Fred's rooms, but he secured quarters in the same quad. He eagerly bought back all the possessions Fred's creditors had claimed—the pictures, books, and bric-à-brac he would never have chosen had they not been Fred's.

Fred's story was still fresh in the public mind when Arthur followed him to the university, and there were many who looked askance at the new Eastgate, as likely to be of the same kidney as his precious cousin; but there were a few who had a sneaking affection for the culprit, and who told him little anecdotes of Fred's brightness and gaiety and good-fellowship. Arthur treasured all these, and wrote home of them to his aunt and sister.

He very soon made a place for himself, and among a better set than poor Fred had chosen. The life could not fail to charm one so vitally endowed with health and spirits and a natural capacity for enjoyment; and if Arthur was not much more inclined in his freshman's term to be studious, and was at all times but a loiterer on learning, he at least won back, during his stay at Oxford, respect for the blemished name of Eastgate.

And meanwhile the other Eastgate, whose brief day of pleasure had ended so disastrously, was paying the penalty in those nine months of terrible silence and solitude with which a convict sets out. What did he think of during those endless weeks, when his lips were sealed, and when, in the loneliness of his cell, all the naked, haggard facts of his life stared him in the face?

For those nine months the same London held father and son; London, with its terribly sharp contrasts, its mocking differences. They lived within a mile or two of each other, nearer than they had often been in their separate lives, but never more widely severed in feeling. Mr. Eastgate told himself that he was childless; and yet, perhaps, the gloomy, harsh old man, who was free to come and go, to surround himself with comforts and luxuries, if he cared for these, was more unhappy than the criminal behind prison bars.

*(To be continued.)*

